# Methods for Synthesis and Extraction of Resveratrol from Grapevine: Challenges and Advances in Compound Identification and Analysis

**DOI:** 10.3390/foods14071091

**Published:** 2025-03-21

**Authors:** Ramona Căpruciu, Constantin Nicolae Gheorghiu

**Affiliations:** Department of Horticulture and Food Science, Faculty of Horticulture, University of Craiova, 13 A.I. Cuza Street, 200585 Craiova, Romania; nicolae.gheorghiu@edu.ucv.ro

**Keywords:** resveratrol, grapevine components, products, by-products, synthesis, extraction, identification, stability

## Abstract

Resveratrol is the most important biopotential phytoalexin of the stilbene group (natural polyphenolic secondary metabolites), synthesized naturally by the action of biotic and abiotic factors on the plant. The yield of individual bioactive compounds isolated from grapevine components, products and by-products is directly dependent on the conditions of the synthesis, extraction and identification techniques used. Modern methods of synthesis and extraction, as well as identification techniques, are centred on the use of non-toxic solvents that have the advantages of the realisation of rapid extractions, maintenance of optimal parameters, and low energy consumption; this is a challenge with promising results for various industrial applications. Actionable advances in identifying and analysing stilbenes consist of techniques for coupling synthesis/extraction/identification methods that have proven accurate, reproducible and efficient. The main challenge remains to keep resveratrol compositionally unaltered while increasing its microbiome solubility and stability as a nutraceutical in the food industry.

## 1. Introduction

**Figure 1:**
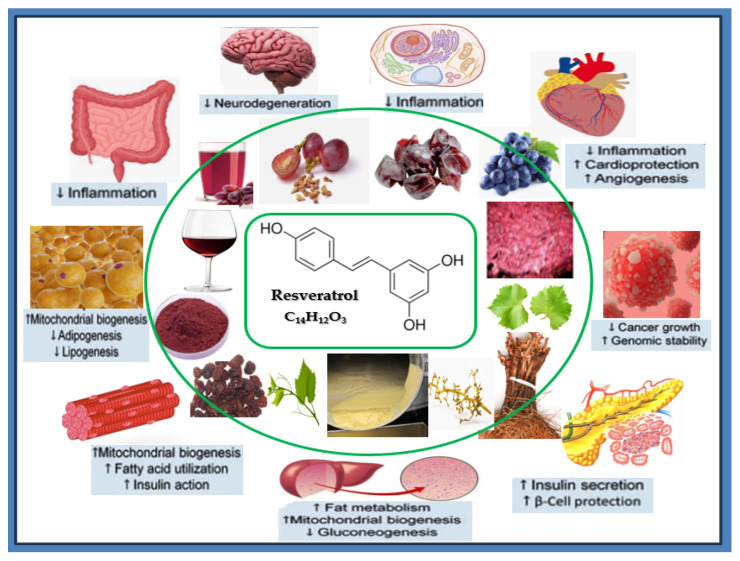
The biological effects of resveratrol from grapevine components, products and by-products.

Originating from grape skins, resveratrol is a phytoalexin, a compound produced by grapevine components that acts similarly to an antibiotic in response to the attack of stressors such as the fungus *Botrytis cinerea* [1,2]. *Cis*- and *trans*-isomer resveratrol occurs both in grapevine components and in products and by-products resulting from applied technologies, with considerable attention in the biomedical literature being given to the *trans*-resveratrol isomer (3,4,5′trihydroxyl-trans-stilbene)—tR. Many publications attest to the existence of resveratrol in grape berries, skin, seeds, pulp, stems, stalks, leaves, vine shoots or roots. Resveratrol is also present in products resulting from the various technologies applied to grapes: wine and juice, grape skin powder, raisins and by-products of the vine: grape pomace, grape canes, wine less and various extracts. Recent studies show that the content of resveratrol is higher in the cut grape pomace than in other produscts (wine, grapes, raisins, etc.) and varies depending on numerous internal and externalfactors, since the applied synthesis and extraction methods have a major role in its quantity and stability [3,4]. Early research such as [5] showed how the concentration of resveratrol during alcoholic fermentation increases in the must and decreases in the skins of black grapes while remaining constant in the seeds. The study shows that after malolactic fermentation, the amount of resveratrol is about twice the amount measured at the end of alcoholic fermentation, indicating a resveratrol amount probably in the form of glucosides or oligomeric form from which the enzymatic activity of malolactic bacteria could release free resveratrol. In recent years, resveratrol is considered a qualitative, integral part of wine due to its several beneficial effects on human health [6]. The identification of resveratrol in grapevine [7] makes this plant of particular importance for industrial, medical and food research [8], and the demand for products based on resveratrol extracted from grapevine components, products and by-products has increased. Numerous types of research prove its beneficial role for health, tackling diseases with an increased incidence: anticancer activity [9], cardioprotection [10], neuroprotection via upregulation of endogenous antioxidant expression and activity [11], protection against diabetes [12] or reducing degenerative effects of neurological diseases such as Alzheimer’s or Parkinson’s [13,14], antioxidant activity [15,16,17], inhibition of platelet aggregation [18] of anti-inflammatory activity [19], etc. (Figure 1).

In recent years, understanding the “French Paradox” has stimulated a new research interest, revealing that the resveratrol synthesized in grapes and contained in wine plays a beneficial role in certain cardiovascular regulatory mechanisms [20,21]. Research in the food industry is interested in using resveratrol in products to increase their functionality [22,23]. Also, maintaining stability after extraction represents a particular interest of current research. The progress that researchers have made so far in the techniques for extraction and identification of resveratrol and, in particular, tR is evident. Thus, research in the last decade has focused on the modernization of synthesis and extraction methods in order to create premises in which the use of low-toxicity substances can prove its efficacy, decreasing the extraction time by increasing the efficiency of extraction methods with reduced energy consumption; however, the challenge of keeping intact the bioactivity of resveratrol is still topical due to its instability. This study presents the most important methods of synthesis and extraction, the progress made by researchers in terms of identification techniques and aspects regarding the use of simultaneous several methods simultaneously (coupling of methods) so that the obtained results can lead to the accurate determination of resveratrol under the most natural conditions, taking into account its current usage trends.

## 2. Methods of Synthesis and Extraction

The methods for synthesizing and extracting resveratrol from grapevine components and products are diverse (chemical, natural, biotechnological), utilizing high-performance technology and high-purity gradients. Recently, alternative solvents (deep eutectic solvents—DESs) have significantly increased the concentration of extracted polyphenols, and the ultrasound-assisted extraction method (UAE), provide higher yields than classical methods. Recent studies show is focused on the use of combined synthesis and extraction methods (chemical, natural and biotechnological) which have proven the efficiency of the process. The current priority criteria in terms of innovative extraction are represented by the preservation of the bioactivity of compounds in natural products through the use of environmentally friendly, efficient nanotechnologies, with the obtaining of extracts with high purity, stable, while preserving the condition of an unaltered environment [24].

### 2.1. Synthesis and Chemical Extraction Methods

The best classical solvents for extracting stilbenes from grapevine cords are alcohols that have a hydroxyl group (MeOH-methanol or C_2_H_5_OH-ethanol) [25]. One of the most well-known methods for the chemical extraction of polyphenols is the MeOH method developed by [26]. Thus, dried grape skin samples (approximately 2 g) and dried seed samples (approximately 1 g) were extracted three times with 20 mL MeOH containing 0.1% HCl (skin) and 10 mL MeOH/H_2_O (80/20) containing 0.1% HCl (seeds). Using the MeOH/H_2_O mixture (70:30, *v*/*v*) as solvent, ref. [27] shows the presence of total polyphenols and RSA in grape seed extracts and grape skin and pulp extracts, with higher potential for seeds.

Also, ref. [28] obtained good results for extracting stilbenes from grapevine compounds using C_2_H_5_OH and MeOH and found that the other stilbenes were better extracted in acetone. The optimization of solvent (water, C_2_H_5_OH, acetone-C_3_H_6_O, MeOH and butanol) extraction on phenolic compounds from grape must based on an experimental design was investigated by [29], who concluded that acetone and C_2_H_5_OH facilitate the extraction of phenolic substances from grape must, and C_2_H_5_OH is more recommended because it is considered an environmentally friendly solvent. Good extraction was obtained with C_2_H_5_OH/H_2_O (80:20, *v*/*v*) by [30], showing that recovery (>96%) and reproducibility (6.83–15.13%) were satisfactory. After extraction, the resveratrol isomers in grape skin were quantified by high-performance liquid chromatography coupled to a visible ultraviolet–visible diode array detector. In order to improve the (endogenous) tR content in grapes harvested at late maturity (LMC), several short anoxic dry nitrogen treatments were applied, and the results allowed the design of an anoxic treatment protocol for grapes prior to the vinification process, which resulted in tR enriched wines [31]. In another study, the skins of red and white grapes were separated from the other GP residues and subjected to extraction with 1:1 C_2_H_5_OH–acidic water as an extractant to obtain as many phenolic compounds as possible from this material [32]. Combined methods to improve synthesis and extraction processes are also used by [33], evaluating the effect of pressure (100, 400 bar), temperature (35, 55 °C) and modifier addition (5% C_2_H_5_OH, *v*/*v*) to identify the optimal extraction of resveratrol from GP obtained as a by-product in winemaking. The remarkable results have been achieved when combining high pressure with low temperature, using 5% C_2_H_5_OH, *v*/*v* as co-solvent.

### 2.2. Synthesis and Natural Extraction Methods

Some of the most important extraction methods applied to grapevine products and by-products, in addition to conventional extraction by maceration (MAC), are represented by sustainable extraction techniques, such as microwave-assisted processes (MAEs), UAE, pressurized supercritical fluids and hydrothermal fluids, in order to obtain safe, stable and high-quality extracts. Traditional extraction methods are energy-intensive and use toxic, expensive, environmentally unfriendly solvents. Although they offer satisfactory extraction yields, they cannot maintain the stability of some heat-sensitive compounds (including resveratrol), hence the need for research to find innovative techniques.

#### 2.2.1. Conventional Extraction (Maceration)

Carrying out dynamic MAC with hydroethanol solution on grape seed powder (30 mL to 1 g of powder sample) followed by simple solid–liquid organic extraction yielded good results (45.7 ± 0.2 mg/g total phenolic compounds extract) in the research by [34]. The combination of cold MAC with thermomaceration (heating crushed grapes at 50 °C for 1 h) and enzymatic macerationperformed by [35] increased the total phenolic compounds content (tR increases from 0.09 to 0.23 mg/100 g). The total phenolic compounds and antioxidant capacity were monitored during conventional fermentation (10 days) by [36]. The main conclusion is that compared to conventional heat treatment, the phenolic compound content must be doubled immediately after OH treatment (ohmic heating) at preset parameters (E = 55 V/cm, t = 60–90 s, T = 72 °C).

Testing the use of an alternative maceration technique (nitrogen maceration) instead of carbonic maceration by [37] resulted in increased polyphenols and anthocyanins in macerated wines. In another study, four environmentally friendly extraction methods were tested, obtaining 29 polyphenols, including stilbene, from grapevine stems, involving the use of polyethylene glycol and water (natural solvents), together with advanced techniques such as low pressure, MAC or UAE, two of which had higher efficacy (water + MAE + UAE + atmospheric pressure (1121 ± 4.8 μg/g tR), water + MAE + UAE + reduced pressure (916 ± 1.9 μg/g tR)), the others yielding lower amounts (694 ± 1.0 μg/g tR) [38]. An interesting approach to the impact of prolonged MAC (6 months) on phenolic quality is found in the study by [39], which shows that this quality is maintained for 4 months, after which a decrease is witnessed probably due to precipitation/reabsorption while the extraction of phenols from the seeds occurred during longer maceration periods, with differences from one variety to another.

#### 2.2.2. Ultrasound-Assisted Extraction (UAE)

UAE has been increasingly used in recent times. It is also referred to as a ‘green’ or environmentally friendly method due to its use as a pre-treatment on plant matrices to facilitate polyphenol extraction. Compared to other extraction methods, the benefits of this method are clear: minimization of the number of solvents used, low execution time and low investment with high yields. The UAE for extracting resveratrol from grapevine cords with three choline chloride-based NaDESs (deep natural solvent systems) was tested [40]. Complementarily, the Box–Behnken Experimental Design (BBD) was applied with water content (5–27.5–50), solid/liquid ratio (10–30–50 mg/500 µL of BCH), temperature (20–50–80 °C) and extraction time (5–32.5–60 min) to improve both polyphenol levels and antioxidant capacity. The study concludes that the optimal extract contains high proportions of stilbene (tR and *trans*-ε-viniferin), demonstrating that NaDESs, as an environmentally friendly alternative to classical organic solvents, are effective for the extraction of resveratrol from cords. Other studies use the Box–Behnken Experimental Design (BBD) and Response Surface Methodology (RSM). The use of BBD combined with RSM to determine the optimization of cold plasma treatment time and voltage on the quality characteristics of non-gold and golden raisins resulted in prolonged shelf life and, at the same time, increased freshness while maintaining the quality parameters during storage [41]. Also, ref. [42] utilized RSM combined with applying PEF, obtaining a significant increase in the extraction yields of biologically active compounds from GP from black grapes. In another study by [43], the impact of temperature, extraction time, solid/liquid (S/L) ratio and mixing speed on extraction efficiency was evaluated using a BBD and response surface modelling. The extracted compounds were evaluated in terms of their physical properties (conductivity, total dissolved solids and pH) and chemical properties (total polyphenol content and antioxidant activity). The BBD on grapevine strings with established parameters (water content (5–27.5–50), solid/liquid ratio (10–30–50 mg/500 µL of 1,4-butanediol—BCH), temperature (20–50–80 °C), extraction time (5–32.5–60 min)) to improve the levels of polyphenols including resveratrol as well as the antioxidant capacity was also applied with good results by [40]. The use of the BBD on GP by [44] yielded the best extraction conditions, resulting in the concentration of phenolic compounds (including tR), anthocyanins and increased antioxidant activity (using ABTS and DPPH assays). Also, ref. [45] identified the antioxidant activity of polyphenolic compounds in the skins and seeds of some grape varieties by performing the ABTS assay; the result obtained in terms of antioxidant activity is positive.

Investigations on the effects of ultrasonic pretreatment (53 kHz, 300 W, 30 °C, 300 s, in a mixture of 5% K_2_CO_3_ + 1% OO solution) and drying air temperatures (40–60 °C) on the drying behaviour, colour values, physicochemical properties (moisture content, VCC, TPC and antioxidant capacity (DDPH)) and the hydration and rehydration capacity of grapes were re-performed by [46]. As a result of the study, it can be said that UAE application can be successfully used to obtain raisins while maintaining a higher degree of nutritional value of grapes compared to classical drying. The sonication (53 kHz frequency at 100% amplitude for 20 min) and thermosensation method was applied by [33] on crushed grape berries, and it was found that compared to the enzymatic technique, tR increased from 0.09 to 0.23 mg/100 g. In order extract polyphenols from grapevine cuttings, in ref. [47], UAE and solid–liquid extraction by means of DES and LA (levulinic acid) were used as an alternative to traditional chemical solvents. Using these methods, the extraction operations identified and quantified eleven polyphenols belonging to the proanthocyanin, stilbene, hydroxycinnamic acid and flavonol families. UAE is characterized as a grapevine stem extract (GSE). Important phenolic compounds were detected as stilbenoids; among them, tR was highlighted. GSE was administered to an animal model of isoproterenol-induced myocardial injury. The extract attenuated the symptoms associated with drug administration: the plasma triglycerides, lipids and total cholesterol, disturbed plasma ion accumulation, markers of cardiac dysfunction and myocardial tissue necrosis were decreased. The phenomenon can be linked to antioxidant capacity of GSE together with its antioxidant capacities, high levels of own antioxidants and the reduction in pathological processes in the lipid sphere in the cardiovascular system [48].

#### 2.2.3. Microwave-Assisted Extraction (MAE)

In the study [44], the environmentally friendly technologies of MAE and UAE were used to recover compounds of interest from the GP. The use of MAE (600 W for 2 min for three cycles) on crushed grapes with the achievement of different maceration temperatures in the product mass, supplemented with sonication, also yielded results in the study of [35], and the tR content had a significant increase when this treatment was applied. Compared with the enzymatic technique, the combined MAE and sonication techniques have led to the detection of more antioxidant polyphenols, such as tR, concluding that the MAE technique was more effective for antioxidant capacity. However, the results of sonication, both for cold and thermosonication, were lower than those of enzymatic treatment [49]. Regardless of the extraction method used (dynamic MAC, UAE and MAE), extracts obtained from GP and seeds showed relatively high concentrations of phenolic compounds [50].

#### 2.2.4. Membrane Extraction

Compared to classical techniques, membrane extraction reduces operating and maintenance costs, keeps temperature and pressure parameters unchanged and obtains superior extracts quantitatively and qualitatively. Its disadvantages include changes in pore geometry and size of constituent molecules [51]. The use of grapevine (*Vitis labruscana*) callus suspension cultures in a conditioned environment (pH, temperature, time, enzyme biocatalyst) and by in vitro bioconversion transforms tR into δ-viniferin, and the proposed method could be a novel approach for in vitro transformation of important molecules [52]. The potential to produce concentrated fractions of bioactive compounds from wine yeast on nanofiltration membranes was studied by [53] following the performance of the three membranes used in terms of productivity, loading index and retention to target compounds (polyphenols, flavonoids, sugars) and antioxidant activity. Thus, the used membranes can be considered suitable for the production of a concentrated fraction of phenolic compounds from wine yeast extracts. The effect of cold plasma treatment on various factors—moisture content (MC), pH, hardness (H), antioxidant activity (AOA), total phenolic content (TPC), rehydration ratio (Rr), browning index (BI) and colour difference (ΔE) —in black raisins and golden raisins were investigated by [41], resulting in improvement in H, Rr, BI, AOA and TPC parameters. Thus, the application of cold plasma treatment can be introduced in food processing due to the prospects of significantly improving food quality by modifying/maintaining physicochemical and nutritional characteristics.

The use of membrane processes in polyphenolic extraction processes are techniques of current research interest.

#### 2.2.5. Supercritical and Pressurized Fluid Extraction (SCFE)

The use of SFE PFE to separate bioactive substances from various resources is a topical issue [22,49,54]. Regarding phenolic compounds (they have polar water-soluble components, thus being highly effective in SCFE), the main advantage of using supercritical and pressurized fluids in resveratrol extraction is the preservation of quality and purity as the process takes place under controlled conditions of light and air, parameters that raise the incidence of degradation reactions. On grape seeds, ref. [55] performed SCFE extractions (temperature 80 °C; CO_2_ flow rate 69 g/min; pressure 250 bar; time 60 min) and found that total polyphenols did not change significantly. The supercritical fluid extraction of polyphenolic compounds from grapevine components has advantages over traditional methods. An example of comparison can be the classical extraction by the SOX method, in which parameters such as light, temperature, pressure and working time cannot be controlled, compared to the SCFE method, which, due to its improved selectivity, speed, versatility, automation and environmental safety, becomes innovative, with qualitative and precise results. The negative aspect of SCFE remains the rather high cost. A possible solution to ensure cost-effectiveness is to use pre-treatment processes of grapes or other vine components to prepare the substrate for SCFE application. These may include advanced shredding precedes (e.g., the grapevine can be crushed and powdered, the skins, seeds, leaves, etc., can be dried and powdered, etc.) to facilitate the application of enzymatic pretreatment, UV-C signals, in combination with the use of MAE or UAE, which can make extraction of biological compounds from the grapevine, including resveratrol, more efficient. Another method involving pre-pressure, temperature and carbon dioxide is based on supercritical extraction and was developed by [56]. They use the supercritical fluid extraction (SFE) technique on dried and ground grape skin powder using a mixture of 90% CO_2_ and 10% ethanol under predetermined temperature and pressure conditions (40 °C and 300 bar). They obtain a green waxy material (11.46%) and a reddish powder (88.54%. On the reddish powder, different extraction processes are further used: Soxhlet (Sox), ultrasonic extraction (USound) and stirring in a tank reactor (Stir).

The application of effective techniques, with good yield, shortening of working time, use of high purity substances, minimal energy consumption, ensuring environmental conservation and, most importantly, from the industrial point of view, preserving the bioactivity of resveratrol, is found in the research of the last decade. Thus, innovative techniques such as PHWE and EHD demonstrate their efficiency in optimizing extraction yields while preserving the biological activity of the target compounds [57,58].

#### 2.2.6. Applying Electric Fields

The use of advanced techniques such as electrospinning to get ultrathin nanofibers and membranes is an innovative method to create nanomaterials with different physico-chemical and biological properties, thus increasing the variability of the fields of use [59]. The application of pulsed electric fields (PEFs) is an effective approach to increase the extraction yield of compounds with biological activity from black (GP) [42], with positive results. The innovative use of electrospun nanofibers with grapevine leaf extract is a novel approach aiming to increase the synergistic biological action of the active compounds present in the extracts, with direct benefits for the development of nutraceutical products [60], attracting more and more interest from the food industry.

#### 2.2.7. Other Methods

Finding different methods to treat grapes in the postharvest period with the possibility of extending their shelf life may lead to variations in resveratrol content. Thus, ref. [61] evaluated the effect of postharvest coating with chitosan—CH 1.0%, ghatti gum—GG 1.0% and combinations (GG 0% + CH 0% (control, distilled water); GG (GG 1.0% + CH 0%); CH (CH 1.0% + GG 0%); CH + GG (GG 1.0% + CH 1.0%) on the nutritional properties, phenolic compounds and antioxidant capacity of ‘Rishbaba’ grapes (*Vitis vinifera* L.) during 60 days of storage at a set temperature and humidity conditions (0 ± 1 °C and 85% relative humidity). During storage, these treatments decreased the resveratrol content from 11.9 μg/g^−1^ FW to 9.4 μg/g^−1^ FW. The only coating that inhibited mould incidence and delayed the changes in resveratrol content was the CH + GG combination, which was considered edible and biodegradable. The up-regulation of phenylalanine ammonia ligase, cinnamate-4-hydroxylase, coumaroyl-CoA ligase and stilbene has a positive and direct relationship with the process of resveratrol synthesis and accumulation [25]. The reduced pressure extraction (RPE) technique is one of the efficient methods for polyphenol extraction, having a dual role (by lowering the extraction temperature, it prevents the deterioration of stilbenes while increasing their purity) [62].

The potential of micellar solutions of nonionic surfactants and poloxamers for the extraction of polyphenols from GP from the vinification of red grapes was studied by [28], resulting in a 19% increase in total polyphenol extracts when using these micellar solutions compared to those obtained by the action of pure surfactants. Also, ref. [63] traced the potential of eleven nonionic surfactants belonging to the poloxamer, Brij, Triton and Tween subgroups with the result that aqueous solutions of nonionic surfactants are efficient media suitable for simple resveratrol extraction.

Resveratrol can also be obtained synthetically in the laboratory by the Heck and Perkin reactions [64], but with low purity, reducing its potential for use in food or medicine.

In order for resveratrol to maintain its bioactivity, strategies such as the application of environmentally friendly extraction with long-term effects, lower energy consumption due to shorter extraction time, use of natural solvents and a combination of deep eutectic solvents (DESs) with environmentally friendly extraction techniques can be used in order to increase and maintain the stability of resveratrol in natural extracts. The combination of DESs and modern extraction techniques has become a growing practice in recent years, given the potential by the increasing demand for resveratrol [8]. Demonstrating the significant potential for extraction and quantification of bioactive compounds from natural products and food, this approach opens new innovative perspectives in the food, pharmaceutical or medical industries, stimulating the extraction of biocompounds by environmentally friendly extraction techniques. Current research focuses on these innovative strategies, making a significant contribution to supporting their application in industrial sectors such as food and pharmaceuticals. Several types of research show the incorporation of various raw materials with bioavailable compounds (e.g., grape pomace powders in food products such as bread [65], pasta [66], ultra-processed products such as hamburgers [67], fortification of bird feed [68], integration into gelatines used in eco-friendly packaging [58], etc.), with promising results. Another approach would be the use of innovative extraction techniques on viticultural food wastes (grape pomace, skins, seeds, wood, etc.) in order to obtain valuable biocompounds with food or pharmaceutical reuse. This can be a permanent strategy in the management of wine-growing areas. Emerging extraction methods are a future approach to produce biologically active compounds from grapevine components, products and by-products for nutraceutical use in industry (food, pharmaceutical, medical).

In the medical sphere, the active implementation in recent years of strategies to increase the stability and bioavailability of resveratrol has led to the highlighting of the positive effects of resveratrol supplementation: it regenerates muscle [69], influences lipid metabolism in obesity [70], impacts oxidative stress markers influencing the incidence of different types of cancer [71,72], improves cardiovascular system function [73] and exhibits neuroprotective [74] and anti-inflammatory [75] capacity. Currently, studies focus on obtaining nanoparticles with resveratrol for better stabilization [76]. The challenges related to the rapid metabolization, low solubility and low bioavailability of resveratrol require further studies for food or clinical applications.

### 2.3. Biotechnological Synthesis and Extraction Methods

**Figure 2:**
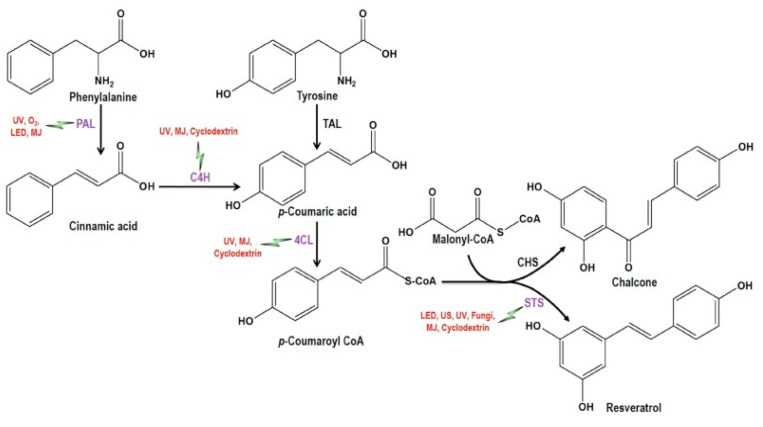
Biosynthesis of resveratrol in grapevine [78].

The biosynthesis of stilbenes in grapevine occurs under the action of an enzyme package. The first step is the oxidative deamidation of L-phenanine to cinnamic acid by phenylalanine ammonia lyase to generate resveratrol [77], which is further metabolized by specific enzymes (phenylalanine/tyrosine ammonia lyase (PAL/TAL), cinnamate 4-hydroxylase (C_4_H), 4-coumarate-CoA ligase (4CL) and stilbene synthase (STS)), resulting in a diversity of biologically active compounds with different characteristics and stabilities (Figure 2). Also, thirteen grapevine enzymes were identified that can utilize resveratrol as a development medium, including ten peroxidases, two glycosyltransferases and one O-methyltransferase [78].

Three possible types of resveratrol hydroxylation enzymatic reactions were tested to reveal hydroxylated resveratrol derivatives, similar to the activity of the polyphenol oxidase cresolase (PPO), with PPO having maximum activity in the crude extract, performed by [79]. The ultimate goal was the detection of piceatannol, a naturally occurring hydroxylated analogue of resveratrol with a higher bioavailability and health-beneficial properties than resveratrol. The hydroxylation of resveratrol to produce piceatannol has also been studied by other investigators, being reported for some human cytochrome-dependent hydroxylases [79,80,81,82] and bacterial cytochromes [83,84]. In grapevine compounds, resveratrol can undergo isomerization processes mainly catalysed by transferases (glycosyltransferases, methyltransferases), hydroxylases and pe-oxidases, resulting in various resveratrol derivatives, with oligomers being the most prevalent [85,86]. The evidence of ACE inhibitory activity is found in the research by [48], which indicates the potential of GSE to ameliorate cardiovascular diseases. Thus, the research shows that not only the singular tR is protective of the cardiac system, but also GSE, through its stilbene and derivative content and improved lipid profile, had an antioxidant role; the extract could be used in the creation of novel ingredients with functional character. Bioenzymatic transformation, cell suspension and bioactivity at the cellular level in plant callus are reactions that can produce stilbenes such as viniferin and resveratrol [52]. To further investigate and study the cell-wall architectural networks present in some grape varieties’ skin and pulp tissues, different carbohydrate-active, enzyme-active treatments were tested by [87], showing the very clear trend of cell-wall degradation in this context. In vitro tests have demonstrated the antifungal activity of several pure stilbenoids, and extracts from annual and multiannual rootstocks have been proposed as a natural alternative to chemical fungicides for sustainable viticulture [88]. The influence of stilbene content in the biotransformed extract obtained from multiyear wood and grapevine roots on Botrytis cinerea attack was studied by [1]. The formation of the active oligomerized stilbene system in the extract, including resveratrol, strongly reduced mycelial growth and spore germination of the fungal agent causing grey mould and also inhibited the production of Botrytis lactazae, despite the ability of the fungus to metabolise some stilbenes. The way how certain pathogens degrade grapevine wood was studied by [4]. This study investigates the presence of the fungi Neofusicoccum parvum and Diplodia seriata on the stump by determining the multitude of existing proteins and enzyme activities carried out outside the cells directly involved in the wood deterioration process and resveratrol metabolisation. It suggests that the activity of pathogenic fungal oxidase could form some resveratrol oligomers present in grapevine wood after pathogen attack.

**Figure 3:**
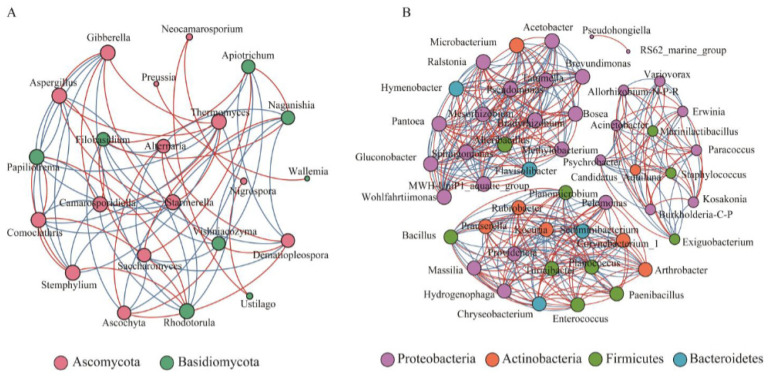
Co-occurrence networks of microbial genera at harvest stage: (**A**) fungi; (**B**) bacteria by [89].

A detailed study of the microbial community at the level of grapes is carried out by [70]. The results show that the total number of endogenous microorganisms changed significantly during the vegetative period of the grapes and was influenced by the growth stage, which can influence resveratrol biosynthesis (Figure 3). The extremes represent the co-occurrence association between microbial genera, with positive and negative correlations coloured in red and blue, respectively [89].

In vitro studies have shown that grape seeds and their extracts inhibit the growth of pathogenic *Enterobacteriaceae bacteria* while leading to the growth and survival of beneficial bacteria, including *Bifidobacterium* spp. and *Lactobacillus* spp. [90].

The biosynthesis of stilbenes is based on specific enzyme packages, and the action of these enzymes by hydroxylation or isomerization reactions on resveratrol leads to the formation of compounds similar in bioavailability and with beneficial properties for human health. The aim of using enzymatic bioconversion, various active enzymatic treatments and plant membranes is to produce resveratrol with high stability, which is imperative in the microbiome and the production of functional food products.

## 3. Identification Techniques

One of the most widely used techniques for the identification of the most important parameters in grapevine components, products and by-products is high-performance liquid chromatography (HPLC) as well as Ultra-High-Performance Liquid Chromatography (UHPLC), and there is a wealth of valuable work of significant research importance. A distinct peak corresponding to the tR reference can be seen in the chromatograph graph.

**Figure 4:**
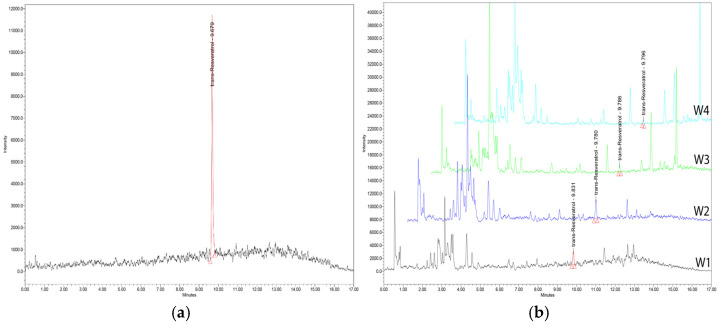
(**a**) Chromatogram of a tR standard showing a prominent peak at 9.67 min, indicative of its purity and concentration in the reference sample; (**b**) overlay of UHPLC chromatograms for Romanian wine samples, with tR peak identified at specific retention time. W1; W2; W3; W4—vine varieties [91].

The UHPLC-MS method thus provides high specificity, ensuring that the observed peak can be confidently attributed to tR without interference from other substances. Such specificity is essential for accurately quantifying complex matrices like wine [91], as shown in Figure 4a. The clean baselines and sharp peak shapes indicate a well-optimized chromatographic method with superior accuracy for sample evaluation and comparison, as shown in Figure 4b.

The resveratrol content in grapevine components, products and by-products can vary and is influenced by many factors (variety, processing methods applied, etc.). Positive results in increasing the content and stability of resveratrol have been observed following the coupling of synthesis, extraction or identification techniques, and these aspects have been the subject of numerous studies recently (Table 1).

As early as [129] showed that fluorimetric detection is much more sensitive than UV detection, and its specificity allows a simple pre-purification of grape berry extracts and direct injection of wines. The RP-HPLC method, described by [130], allows the separation of several types of phenolic compounds present in grapes and wines by directly injecting samples using a binary gradient with salt-free solvents and photodiode array detection. tR was separated on the Nucleosil C18 column with acetonitrile: water (40:60, *v*/*v*) mobile phase, read at 306 nm (UV) at a flow rate of 0.3 mL/min [92]. The concentrations of tR were evaluated using HPLC-DAD in red wines. Ref. [131] observed that during MAC, the maximum extraction of tR was reached after 12 days, after which a decline was observed. Another method with positive results for quantifying free cR- and tR is HPLC coupling (binary gradient) with fluorescence detection [93]. The results for grapes (7–24 mg/L) indicate that wines made from grapes may contain significant amounts of tR. In addition, ref. [132] details the composition of phenols (anthocyanins, flavonols, hydroxycinnamic acid derivatives, stilbene and fla-van-3-ols) in the skin and pulp of seedless table grapes (BRS Clara and BRS Morena varieties) using HPLC-DAD-ESI-MS/MS. These results suggest that the entire grapes, including the skin, may potentially possess beneficial properties to human health; the BRS Morena grape can be considered a high resveratrol producer. The identification of resveratrol was carried in some grape varieties using the HPLC-DAD technique, after fractionation of tR through a 500 mg C18 column (SPI—Solid Phase Isolation technique). A continuous decrease in tR content was observed in all cultivars during ripening [133]. In their study, ref. [34] determined the phenolic profile of grape seeds by liquid chromatography (Dionex Ultimate 3000 UPLC, Thermo Scientific, San Jose, CA, USA) with a diode array detector (wavelengths of 280, 330 and 370 nm) equipped with an ESI source. In another study, ref. [134] identified an appreciable amount of phenolic compounds in raisins and established that raisins are an important source of polyphenols and that there may be significant differences between species or cultivars. Then, ref. [44] performed similar research for GP under similar conditions. An HPLC system (Waters, Milford, MA, USA) was used for polyphenol analysis, by means of which polyphenols such as querce-tin-3-O-glucoside, 5-O-caffeoylquinic acid, cyanidin-3-O-glucoside and resveratrol were identified in GP [47]. High-performance liquid chromatography (HPLC) chromatograms showed that the highest concentration of tR in grape skins was detected in the early period of the ripening stage [135]. The identification and determination of several phenolic groups in grape pulp using HPLC-DAD by [136] showed that there is a close relationship between the accumulation of total or individual phenolic compounds and the extraction method. Using high-performance liquid chromatography coupled to electrospray ionization mass spectrometry/mass spectrometry (HPLC-ESI-MS/MS), ref. [38] identified 29 polyphenolic substances, including resveratrol. By means of LC-ESI-QTOF-QTOF-MS/MS, ref. [95] identified 78 phenolic compounds consisting of flavonoids (36), phenolic acids (31), lignans (3), stilbene (Resveratrol 5-O-glucoside) and other polyphenols (7) in five grape samples. The use of UHPLC has found its applicability for the analysis of phenolic compounds in grape samples, the method being of great interest, among others, because it allows the phenolic characterization of grape varieties accurately in a short time [137]. The quantification of stilbenoids in powder from grapevine cork powder was performed with the UHPLC system at λ 306. HPLC-ESI-MS was used to qualify and identify tR peaks [122].

The identification techniques are diverse, high-throughput and capable of identifying and quantifying an increasing number of polyphenolic compounds, including resveratrol. The couplings between techniques are of interest, increasing the accuracy of phenolic characterization in grapevine components, products and by-products, with shorter turnaround times. At the same time, separation and identification techniques are under continuous innovation to find the optimal solution to increase the stability of resveratrol.

## 4. Challenges and Future Perspectives

In recent years, several schemes have been devised for the natural synthesis of resveratrol, including the use of yeasts [138] or engineering of bacteria or modified plants to provide a constant supply of resveratrol [78]. Although it has high bioactivity, the stability of resveratrol is influenced by internal factors (structure) and external factors (light, oxygen in the air, variable pH and high temperature) [139]. Implementation strategies are converging towards a considerable reduction in energy consumption, using high-purity substances in extraction and prioritizing environmentally friendly and efficient strategies. These aspects have made possible the shift from traditional to innovative extraction in the last decade, primarily due to the detailed knowledge of the profile of substances in the extract, but also due to stricter environmental legislation with enforcement levers recognized at national, European and global levels, as well as due to the trend of increasing consumer demand in the food industry for products as natural, safe, high-functionality and nutraceutical pharmaceuticals, involving green nanotechnology [140], important in improving and maintaining human health. Therefore, these parameters lead to higher quantitative and qualitative yields with enhanced purity, bioavailability and stability of resveratrol, leading to better incorporation into food, pharmaceutical, agricultural or medical composites. Current research brings to the attention of the consumer innovative techniques such as encapsulation in liposomes (nanoparticles) or emulsions [111], combining it with natural compounds with which it does not react (quercitin), achieving structural modifications by applying enzymatic reactions or ecological techniques [141], etc. Whether incorporated in food, food supplements or medicine, the role of resveratrol is to improve human health, as evidenced by the numerous research studies in these fields. Hence, there is interest from researchers and manufacturers to ensure its bioavailability and stability so that consumers can benefit from its properties, given its high availability in the plant kingdom. Currently, conventional extraction (MAC) is being successfully replaced by innovative techniques such as (UAE, MAE, membrane extraction, SCFE, SFE, PEF, etc.). The future is represented by combinations of efficient extraction/synthesis/identification methods based on the use of non-toxic solvents (UAE + BBD, UAE + NaDES + RSM, RSM + PEF, UAE + DES-LA, MAE + UAE + HPLC-GC/MS, MAE + HPLC-DAD(UV)/CAD, UAE+ UHPLC-Orbitrap MS4, UAE + LC-ESI-QToF-MS/MS, etc.).

## 5. Conclusions

Innovative synthesis, extraction and identification techniques offer significant advantages over traditional methods, including low energy consumption, minimal environmental impact and the ability to extract and stabilize the bioactivity of target compounds. Implementing these advantages aims to develop green strategies aligned with national, global or globally aligned environmental standards. Research on resveratrol over the last decade in terms of its synthesis, extraction and identification in order to increase its bioavailability in grapevine components and, at the same time, to enrich products and by-products obtained from the applied technologies with tR, is focused on the technique of combining methods. The results of the combination of synthesis, extraction or identification techniques indicate potential applications on grapevine components, products and by-products as a source of phenolic compounds which can be directed to the food industry as antioxidants, nutraceuticals, activators of ripening processes or food colorants.

In the future, the development and validation of rapid separation methods for the characterization of polyphenolic fractions may lead to increased stability of resveratrol in grapevine components, products and by-products for use in various industrial applications such as functional food, pharmaceuticals, cosmetics, medical products, soil and plant bio-fertilizers, animal feed fortification, bioenergy or biofuel. Some of the ideas and practices developed and implemented in current research have the potential to contribute to industrial development and, at the same time, to improve quality of life.

## Figures and Tables

**Table 1:** Data on the identification of resveratrol in grapevine components, products and by-products.

Fractions		Methods/ Extracting Substances	Detection Technique *	Content in Resveratrol	References
Grapevine components	Whole grape	C_2_H_3_N/H_2_O (40:60, *v*/*v*)	HPLC-UV	0.09235 mg/L, DW	[92]
		C_2_H_3_N–CH_3_COOH	HPLC-FL	7–24 mg/L, DW	[93]
		acidified water (0.1% H3PO4)/C_2_H_3_N	HPLC-GC/MS	13.9 ± 2.87 mg/L, DW	[94]
		70% C_2_H_5_OH	LC-ESI-QToF-MS/MS	227,000 mg/L, FW	[95]
		MeOH (70%)/H_2_O (8:2, *v*/*v*)	HPLC-DAD-ESI-MSn	4.04 mg/L, DW	[96]
	Skin	MeOH	HPLC-ESI-MS/MS	30.6 ± 1.7 mg/L, DW	[97]
		1% HCl in MeOH	HPLC	3.13 ± 0.33 to 14.57 ± 1.34 mg/L, FW	[98]
		incubation time—24 h, US application method-(P01), US frequency—20 kHz, US treatment time—60 min and ultrasonic intensity (UI)—1.15 W cm^−2^	HPLC	180 ± 10 mg/L to 3580 ± 80 mg/L, DW	[99]
		MeOH–deionized water (1:1) with 1% CH_2_O_2_ (*v*/*v*)	UHPLC	0.05 mg/L, FW	[100]
		MeOH	HPLC	0.065 to 7.119 mg/L, DW (*cis*-resveratrol-cR) 0.633 to 9.152 mg/L, DW (tR)	[101]
		MeOH/C_4_H_8_O_2_ (1:1, *v*/*v*)	HPLC	0.667 mg/L, DW	[102]
		70% MeOH	UPLC-MS-MS	2.76 mg/L, FW	[103]
	Seed	MeOH	HPLC-ESI-MS/MS	20.4 ± 0.7 mg/L, DW	[97]
		H_2_O-CH_2_O_2_-C_2_H_3_N (76.935/0.065/23, *v*/*v*/*v*)	UHPLC-MS/MS	305.98 ± 0.23 mg/L, DW	[104]
	Pulp	MeOH	HPLC-UV	45 to 1018.9 mg/L, DW	[105]
	Stem	C_2_H_5_OH (5%, *v*/*v*)	HPLC	680 to 1870 mg/L, DW	[33]
		1. (H_2_O + MAE + UAE + atmospheric pressure); 2. (H_2_O + MAE + UAE + reduced pressure).	HPLC-ESI-MS/MS	1121 ± 4.8 mg/L, DW	[38]
	Leaf	MeOH	HPLC-ESI-MS/MS	6.2 ± 0.1 mg/L, DW	[97]
		The DoE technique applied to red vine leaf c (50% MeOH, temperature 70 °C and three replicates per one hour)	HPLC	0.306 ± 0.009 mg/L DW	[60]
		Two dark sonication replicates two dark sonication cycles (10 mL of 0.1 m HCl 80% MeOH solution) (10 mL of 0.1 m HCl 80% MeOH solution, at 4 °C in 15 min)	UPLC	30–40 mg/L FW^−1^ × 10^−1^	[106]
		UV-C treatment/MeOH	LC-MS/MS	0.01997718–0.3578911798 mg/L, FW	[107]
		70% MeOH	UPLC-MS-MS	4.22 mg/L, FW	[103]
	Shoot	EC50 Caco-2 /EC50 HepG_2_-H_2_O_2_	HPLC	14.74 and 29.47 mg/L, DW	[108]
		MeOH-H_2_O (80:20, *v*/*v*)	HPLC	148.53 mg/L^−1^, DW	[47]
	Root	MeOH	HPLC-ESI-MS/MS	86.3 ± 2.5 mg/L, DW	[97]
		COSMO-RS-NADES	UHPLC-UV	520–2470 mg/L, DW	[109]
	Wood	*Botrytis cinerea* secretome	UHPLC-UV-DAD-MS	9541 ± 16,800 mg/L, DW	[1]
	Woody tissues	80% MeOH	UPLC-MS	69.1 to 436.5 mg/L, DW^−1^	[110]
	Bud	80% MeOH	UPLC-MS	150 mg/L, DW^−1^	[110]
Grapes product	Wine	C_2_H_3_N/H_2_O (40:60, *v*/*v*)	HPLC-UV	0.1047 mg/L, DW	[97]
		MeOH	UHPLC-Orbitrap MS4	4.00 mg/L, DW (red wine)	[111]
		Transepithelial diffusion	LC-MS	0–1.089 mg/L, FW (white wine); 0.29 mg/L, FW (rosé wine); 0.361–1.972 mg/L, FW (red wine).	[112]
		MeOH	UHPLC- MS/MS	0.07–2.61 mg/L, DW (cR) 0.05–3.82 mg/L, DW (tR)	[113]
	Juice	C_2_H_3_N/H_2_O (40:60, *v*/*v*)	HPLC-UV	0.000091 mg/L, DW	[102]
		C_2_H_6_O/water solution (60:40, *v*/*v*)	HPLC	4.4 to 7.0 mg/L, DW	[114]
	Concentrated juice	C_2_H_6_O/water solution (60:40, *v*/*v*)	HPLC	12.4 to 21.3 mg/L, DW	[114]
	Grape skin powder	C_2_H_6_O/H_2_O (50%, *v*/*v*)	GSP/UV-A/HPLC	250 mg/L, DW	[115]
	Raisin	HCl/MeOH/H_2_O, 1:80:19, *v*/*v*/*v*)	UPLC-VION-IMS-QToF	16,544,000 ± 44,000 mg/L, DW	[116]
	Jam	UP200S ultrasonic system optimized with solvent composition (10–70% and 30–90% MeOH in H_2_O; solvent-to-solid ratio (10:1–40:1); ultrasonic probe diameter	UPLC-FD	0.027 ± 0.01 to 1.760 ± 0.04 mg/L, DW	[117]
	Marmalade	BBD optimized with solvent composition (60–100% and 10–70% MeOH in H_2_O); MAE power (250–750 W); solvent-to-solid ratio (20:5–60:5)	UHPLC-FD	1.74 mg/L^−1^, DW	[118]
By-products	Grape canes	C_2_H_6_O/H_2_O (80:20, *v*/*v*)	HPLC-DAD-Q-ToF	227.07 mg/L^−1^, DW	[119]
		The microencapsulation (by spray drying) using maltodextrin (MD) (10% *w*/*v*) and UV irradiation (254 nm)	HPLC	679.6 ± 51.6 mg/L, DW	[120]
		Sonicate/macerate 96% C_2_H_6_O (*v*/*v*)	HPLC-MS	815.9 ± 153 mg/L, DW	[121]
		NADES evaluation combined with HPCC biphasic solvent by COSMO-RS calculations	UHPLC-UV	1.50 mg/L, DW	[122]
		HPLC-UV-DAD	HPLC-ESI/MS	890 ± 20 mg/L^−1^, DW (dormant bud) 610 ± 10 mg/L^−1^, DW (second extended leaf) 200 ± 70 mg/L^−1^, DW (sixth extended leaf and visible inflorescence)	[123]
	Grape pomace	C_2_H_6_O (5%, *v*/*v*)	HPLC	190 to 1073 mg/L, DW	[33]
		Extracted by SOX and MAC in IPA	HPLC-DAD/MS	0.042–0.653 mg/L, DW (tR) 0.05–0.35 mg/L, DW (cRl)	[124]
		UAE for one hour (80% MeOH solution (100 mL) acidified with 0.1% CH_2_O_2_)	HPLC/DAD/T_O_F	100 ± 20 mg/L, DW	[125]
	Wine lees	Conventional aqueous (CE) and non-conventional UAE	HPLC	36,360 mg/L, DW	[126]
		Enzyme-assisted extraction based on the hydrolysis of WL proteins	UHPLC-(ESI+)-Q-ToF-MS	164.00 ± 0.80 mg/L, DW	[127]
	Grapevine extracts	MeOH/H_2_O (50:50, *v*/*v*)	HPLC-DAD(UV)/CAD	36.75 mg/L^−1^, DW (CAD) 211.25 mg/L^−1^, DW (DAD/UV)	[128]

* Abbreviations.

## Data Availability

No new data were created or analyzed in this study. Data sharing is not applicable to this article.

## Abbreviations

The following abbreviations are used in this manuscript:

HPLC	High-performance liquid chromatography
HPLC-MS	HPLC–mass spectrometry
HPLC-UV	HPLC–ultraviolet
HPLC-GC/MS	HPLC–gas spectrometry/mass spectrometry
HPLC-DAD(UV)/CAD	HPLC–diode array detection (ultraviolet)/charged aerosol detector
HPLC-ESI-MS/MS	HPLC–electrospray ionization–mass spectrometry–mass spectrometry/mass spectrometry
HPLC-DAD-ESI-MSn	HPLC–diode array detection–electrospray ionization–mass spectrometry
HPLC-DAD-QToF	HPLC–diode array detection–quadrupole–time of flight mass spectrometry
UHPLC	Ultra-high-performance liquid chromatography
UPLC-FD	UPLC–fluorescence detection
UPLC-MS	UPLC–mass spectrometry
UHPLC-UV	UHPLC–ultraviolet
UHPLC-UV-DAD-MS	UHPLC–ultraviolet–diode array detection–mass spectrometry
UHPLC-(ESI+)-QToF-MS	UHPLC–(electrospray ionization-+)–quadrupole–time of flight mass spectrometry
UHPLC-Orbitrap MS4	UHPLC–Orbitrap mass spectrometry
UPLC-VION-IMS-QToF	UPLC-VION-IMS–quadrupole–time of flight mass spectrometry
LC-MS	Liquid chromatography–mass spectrometry
LC-ESI-QToF-MS/MS	Liquid chromatography–electrospray ionization–quadrupole–time of flight mass spectrometry/mass spectrometry
GSP/UV-A/HPLC	GSP/ultraviolet-A/HPLC
DW	Dry weight
FW	Fresh weight
SOX	Soxhlet extraction
DoE	Design of experiment
RSA	Radical scavenging activity
ACE	Angiotensin-converting enzyme
VCC	Vitamin C
TPC	The phenolic content
IPA	Isopropyl alcohol
cR	*cis*-resveratrol
tR	*trans*-resveratrol
HLM	Harvested at late maturity
PHWE	Pressurized hot water extraction
EHDs	Electrohydrodynamic methods
MAEs	Microwave-assisted processes
UAE	Ultrasound-assisted extraction method
DESs	Deep eutectic solvents
NaDESs	Deep natural solvent systems
